# Type 2 Diabetes Risk among Asian Indians in the US: A Pilot Study

**DOI:** 10.1155/2013/492893

**Published:** 2013-07-22

**Authors:** Annie Thomas, Alyce Ashcraft

**Affiliations:** ^1^Marcella Niehoff School of Nursing, Loyola University Chicago, 1032 W. Sheridan Road, Chicago, IL 60626, USA; ^2^School of Nursing, Texas Tech University Health Sciences Center, 3601 4th Street, Lubbock, TX 79430, USA

## Abstract

The purpose of this pilot study was to investigate type 2 diabetes risk among Asian Indians of Kerala ethnicity living in a West Texas County of the USA. The study used a descriptive correlational design with thirty-seven adult nondiabetic Asian Indian subjects between 20 and 70 years of age. The measurement included nonbiochemical indices of obesity, family history of type 2 diabetes, length of immigration in the US, history of hypertension, physical activity pattern, and fruit and vegetable intake. The majority of the subjects showed an increased nonbiochemical indices corresponding with overweight and obesity, placing them at risk for type 2 diabetes and associated cardiovascular complications. The physical activity pattern indicated a sedentary lifestyle. The decreased physical activity was associated with a higher Body Mass Index (BMI) and body fat percentage; length of residence in the US greater than 10 years was associated with increased body fat percentage and BMI; family history of type 2 diabetes was associated with an increase in body fat percentage. Fruit and vegetable intake pattern was not associated with a risk for type 2 diabetes. Further studies are recommended for risk surveillance among Asian Indian population living in the US.

## 1. Introduction

The prevalence of type 2 diabetes is on the rise globally and has reached epidemic proportions in many countries. The number of adults affected by the disease in 2011 was 366 million, which is projected to increase to 552 million by 2030 [1]. An estimated 25.6 million Americans (11.3% of the population) have type 2 diabetes, and 1.9 million more adults are diagnosed with the disorder each year [2]. Type 2 diabetes is the fifth leading cause of death in the Asian American population. Among Asian Americans, Asian Indians have the highest prevalence of type 2 diabetes compared to other Asian subgroups. Based on the American Community Survey, between 2000 and 2010, the Asian Indian population in the USA grew by 67.60% (3.2 million) and represented the third largest Asian subgroup in the US [3]. Asian Indians who immigrated to Western countries are found to be at risk for the development of type 2 diabetes due to the metabolic impact of a westernized diet or reasons based on tissue resistance to insulin [4, 5]. Asian Indians living in India also face a similar threat related to type 2 diabetes. It is estimated that in another 20 years, nearly one fifth of world's diabetic population will be in India. Asian Indians are more insulin resistant and hyperinsulinemic than whites which puts them at increased risk for diabetes and heart disease despite the absence of traditional risk factors and decreased body weight [6–8].

Epidemiological studies have shown that type 2 diabetes has a global distribution, and its prevalence varies from country to country, in different ethnic groups in the same country, and between the same ethnic groups undergoing internal or external migration. Populations with acculturation from traditional to modern life style have a higher prevalence of type 2 diabetes, with the prevalence of type 2 diabetes in Asian Indians greater than 20 years old being higher than all other racial groups in the US [9–11]. A recent research study focusing on Asian Indians from the state of Michigan found a high prevalence of self-reported type 2 diabetes (20.1%) and elevated/abnormal A1C levels (A1C ≥ 6.5%: 22.6%). It exceeded the percentages of other ethnic groups reported in the 2006–2008 Michigan Behavioral Risk Factor Survey (MBRFS) (8% for non-Hispanic whites, 13% for non-Hispanic blacks, 8.9% for Hispanic Latinos, and 15.6% for Native American/Alaskan natives) [12].

### 1.1. Background

Type 2 diabetes has been associated with reduced physical activity and seems to affect the risk of type 2 diabetes independent of diet. The level of physical activity is higher in ethnic groups living in their countries of origin as compared to the same ethnic groups living in the US [13–15]. Goel et al. [16] conducted a study to estimate the prevalence of obesity among US immigrant subgroups. The study found that the obesity pattern increased by the duration of stay in the US as evidenced by higher Body Mass Index (BMI) rate beginning after 10 years. The prevalence of obesity among immigrants living in the USA for 15 years approached that of US-born individuals. The study recommended early intervention with diet and physical activity which may represent an opportunity to prevent weight gain, obesity, and obesity-related chronic illnesses.

The National Health Interview Survey conducted during 1997–2008 on trends in the prevalence of type 2 diabetes reported two important determinants of obesity and type 2 diabetes in Asian immigrants: age at arrival and period of stay in the US. Findings indicated that the rise in obesity was higher during the first five years of stay in the US from the time of immigration. The prevalence of obesity and type 2 diabetes was greater for those who arrived at a relatively young age. Asian Indians had the highest prevalence of type 2 diabetes, followed by Filipinos, other Asians, and Chinese [4].

Family history has been shown to be a risk factor of number of diseases including type 2 diabetes. Family history, by itself, is most useful in predicting disease when multiple family members are affected. Family history of any specific disease reflects the consequences of genetic susceptibility, shared environment, and common behaviors [17]. In a study investigating the family history of type 2 diabetes and the prevalence of metabolic syndrome in adult Asian Indians, the family history of type 2 diabetes had significant effect on individuals compared to individuals having no history of type 2 diabetes. The study recommended using family history of type 2 diabetes as a predictive tool for early diagnosis and prevention of metabolic syndrome in Asian Indian population [18].

Recent studies have shown that type 2 diabetes can be prevented in high-risk subjects with impaired glucose tolerance by life style intervention. Consumption of fruits, berries, and vegetables, the quantity and quality of dietary fat intake, fiber intake, and physical activity at work and/or on leisure time have been demonstrated to modify the risk of type 2 diabetes [16, 19].

Type 2 diabetes and high blood pressure are strongly linked with each other. High blood pressure can increase the progression of prediabetes into type 2 diabetes and cardiovascular complications [20]. A study conducted by Francis et al. examined risk factors affecting the progression to type 2 diabetes [21]. Individuals with hypertension were more likely to progress from prediabetes to type 2 diabetes. Individuals with type 2 diabetes have increased cardiovascular disease risk compared with those without type 2 diabetes [22]. It has an insidious onset with a long, latent, asymptomatic phase. The prediabetic stages also carry high risk for cardiovascular diseases (CVDs) and clustering of the cardiovascular risk factors [23]. Macrovascular complications are the most important causes of morbidity, mortality, and disability in people with type 2 diabetes. Asian Indians exhibit the highest ethnic-specific prevalence of CVD, with two to three times higher instances than among Caucasians. Asian Indians are at greater risk of developing coronary heart disease compared to other Asian subgroups [24]. 

Asian Americans are sometimes stereotyped as the “healthy minority.” Grouping Asian Americans into a single category hides the health risks of specific subgroups in the US [8]. They face some of the same limitations to good health as other minority groups. Language barriers interfere with receiving quality health care. Many Asian Americans do not know about the risk factors for disease or the role of preventive health care [25]. The cultural beliefs about health and illness often conflict with Western medicine, which keep some Asian Americans from seeking help for symptoms. Screening for type 2 diabetes risk and preventative measures has the potential to improve the health status of this minority population in the US [26].

Asian Indians living in the US are diverse. Cultural beliefs and practices vary from state to state in India and include dialect and religion as well as dietary habits (strict vegetarian versus nonvegetarian) [12]. The present study focused on Asian Indians from the Kerala state of India and living in the US. Kerala Indians represent a broader Asian Indian population in the US. Data are unavailable to indicate the percentages of Kerala Asian Indians living in the US. Given that limited studies have investigated the risk for type 2 diabetes among Asian Indians, it is necessary to describe the risk factors among Asian Indians and their subgroups living in the US. Such data would then be useful in planning a primary prevention program. 

### 1.2. Study Aims

The aims of this pilot study were to (a) describe type 2 diabetes risk among Asian Indian adults of Kerala ethnicity living in the US and (b) to determine the relationship/association between physical activity, length of immigration, family history of type 2 diabetes, and fruit and vegetable intake with the anthropometric measures and body fat percentage.

## 2. Conceptual Framework

The Social Learning Theory and Health Belief Model [27] provided a conceptual framework to guide the study. The theory accounts for individual beliefs about contracting a disease and its potential effects on quality of life. The health belief model theorizes that people are not likely to take action for their health action unless (a) they are susceptible to the disease in question; (b) they believe that the disease would have serious effects on their lives, if they should contract it; (c) they are aware of certain actions that can be taken and believe that these actions may reduce the likelihood of contracting the disease or reduce the severity of it; (d) they believe that the threat to them of taking the action is not as great as the threat of the disease itself; and (e) they believe that they possess the ability to do things on their own. This framework provided the conceptual basis for this study. Screening for type 2 diabetes risk and the emergence of risk variables informs individuals that are susceptible to developing a serious health problem. When it is believed that the occurrence of type 2 diabetes will seriously change lifestyle and taking action could reduce the threat, a person is more likely to engage in preventative measures. Self-efficacy measures and preventative interventions directed at behavior modifications will prevent or control type 2 diabetes among American Asian Indian population.

## 3. Methods

### 3.1. Design and Subjects

A descriptive correlational design was used for this pilot study. The subjects included a convenience sample of 37 Asian Indian adults of Kerala ethnicity attending a church in Lubbock County, TX, USA. The inclusion criteria included the following: (a) Asian Indians of Kerala origin, (b) no known history of diabetes (type 1, type 2, or gestational), (c) from 20 to 70 years of age, (d) living in the US for more than a year, and (e) ability to speak and read English. Exclusion criteria included the following: (a) documented history of diabetes including gestational diabetes, (b) pregnancy, and (c) history of implanted defibrillator (the foot sensor pads on the Tanita scale sends a low electrical signal through the body for measuring the body fat percentage which can alter the implantable defibrillator settings). The subjects were recruited for the study through advertisement using flyers. 

### 3.2. Ethical Considerations

The university's institutional review board approved the research procedures for the recruitment and data collection. All participants enrolled in the study spoke and read English. The purpose of the research was described in detail prior to obtaining written informed consent from all participants. 

### 3.3. Data Collection

Data were collected from all the participants during a twelve-week time period. It took approximately one hour to explain the consent details and to record the measurements from each subject. Participants returned the completed questionnaires the following week of the measurement procedures. The following measures were used to collect the data.

#### 3.3.1. Demographic Data

Demographic data included age, gender, religious preference, food preference, length of residence in the US, family history of diabetes, and history of hypertension. The reference values for high blood pressure were systolic blood pressure greater than or equal to 140 mmHg or diastolic BP greater than or equal to 90 mmHg (adapted from the Centers for Disease Control (CDC)) [20]. The data on high blood pressure were obtained using an investigator developed self-report questionnaire. 

#### 3.3.2. Anthropometric Measures and Body Fat Percentage

Anthropometric measures included the following: height (measured by a Stadiometer using a centimeter scale), weight (measured by a Tanita scale), waist circumference (WC) (measured by a nonstretchable tape midway between the costal margins and the iliac crests with participants standing erect, at the end of normal expiration), hip circumference (measured around the widest part at the level of greater trochanters), and sagittal abdominal diameter (SAD) (measured by using Holtain Khan's sliding abdominal caliper with parallel blades with the subjects in supine position, hips flexed with the maximum diameter of the abdomen in sagittal plane).

The body fat percentage was measured through bioelectrical impedance analysis using a Tanita scale (Tanita's Patented BIA method). After entering the age, gender, and height, the subject was instructed to step on to the platform of the Tanita scale. Electrodes in the foot sensor pads send a low, safe signal through the body. Weight was calculated automatically along with body fat content in less than minute. The body fat percentage and anthropometric measures were obtained by experienced research personnel to ensure consistency with the data collection. 

The reference values for the BMI were between 23–25 kg/m^2^ and >25 kg/m^2^ to delineate between overweight versus obesity. These values are recommended by the World Health Organization Western Pacific Region and International Obesity and International Association for the Study of Obesity for Asian populations [28].

 The reference values for the SAD, WC, waist-hip ratio (WHR), and body fat percentage for the present study are selected based on the recommendations from the Chennai Urban Rural Epidemiology (CURE) Study [29], Indian Urban Based Study [30], and World Health Organization [28] recommendations. The reference values for WC were 87 cm and 82 cm for men and women, the WHR were 0.88 and 0.81, and the SAD was ≥21.5 cm. The reference value for the body fat percentage was ≥25%. A gender specific reference value was not available for SAD and body fat percentage measures. 

#### 3.3.3. Global Physical Activity Questionnaire

The Global Physical Activity Questionnaire (GPAQ) is a standardized 16-item questionnaire developed by the World Health Organization [31]. GPAQ is a previously validated and accepted measure of physical activity. A nine country reliability and validity study of the GPAQ revealed that the reliability coefficients were of moderate to substantial strength (Kappa = 0.67–0.73, Spearman rho = 0.67–0.87). The criterion validity and content validity correlations of the GPAQ were 0.35 and 0.65 [32], respectively. The physical activity questionnaire includes the following physical activity patterns: activity at work, travel to and from places, recreational activities, and sedentary behavior. Levels of total physical activity were measured as metabolic equivalent tasks per week (METs/week). The amount of energy expended for different activities varies with the intensity and type of exercise. One MET is the energy expended at rest, two METs indicate the energy expended is twice that at rest, three METs are triple the resting energy expenditure, and so forth. To get weekly MET scores, MET values for each activity are multiplied by the hours expended in that activity each time then added. The subjects in the study were categorized as “physically inactive” if the METs/week were <3000 [31].

#### 3.3.4. Food Frequency Questionnaire

The Food Frequency Questionnaire (FFQ) is a standardized 35-item questionnaire adapted from John Hopkins Weight Management Center. This questionnaire is used to assess overall dietary intake including fruits and vegetables. The reliability and validity of the FFQ has not been reported in the literature. This was one of the limitations of this pilot study. Two previous studies linked the consumption of fruits and vegetables less than 3-4 times/week with type 2 diabetes risk [9, 19]. Therefore, the present study measures only the fruits and vegetable consumption from the food frequency questionnaire.

### 3.4. Data Analysis

Descriptive statistics were used to analyze the demographic variables (age, gender, immigration period, family history of type 2 diabetes, and history of hypertension) and other type 2 diabetes risk variables (anthropometric measures, body fat percentage, physical inactivity, and fruits and vegetable intake). A Pearson's correlation analysis (*r*) was used to examine the relationship of physical activity patterns with anthropometric measures of obesity and body fat percentage. *χ* ^2^ test and Cramer's *V*  (*V*) were used to examine the degree of association of categorical variables (family history of type 2 diabetes, immigration time in the US, and fruit and vegetable intake) with the anthropometric measures and body fat percentage. The correlations/associations are determined statistically significant at *P* value less than or equal to 0.05 (*P* ≤ 0.05). Data analysis was performed using SPSS software (version 14.0) (Cary, NC, USA).

## 4. Results

### 4.1. Description of Demographic/Type 2 Diabetes Risk Variables

#### 4.1.1. Demographics

Demographic data is presented in Table 1. The sample consisted of 21 (56.76%) males and 16 (43.24%) females. The mean age was 38.24 (SD = 16.24) with majority in the age group of 20–29 (13, 35.12%) and 30–39 (13, 35.12%). The religious preference for all subjects in the study was Christian. The food preference was nonvegetarian for the majority of subjects (33, 89.18%). The length of immigration time in the US was between 1–5 years for 17 (45.94%) subjects, 5–10 years for 5 (13.54%), and >10 years for the other 15 (40.52%) subjects. Twenty-one subjects (56.82%) reported a family history of type 2 diabetes. Only 9 subjects reported hypertension, and most (*n* = 28, 75.75%) had a blood pressure value of <120/82 mmHg taken during the past one month before the study.

#### 4.1.2. Anthropometric Measures and Body Fat Percentage

Anthropometric measures and body fat percentage data are presented in Table 2. The majority of subjects (*n* = 32, 86.48%) had a BMI ≥ 23 kg/m^2^, of which 14 (37.83%) subjects had a BMI between 23 and 25 kg/m^2^, and 18 (48.64%) subjects had a BMI > 25 kg/m^2^. The mean BMI was 26.19 kg/m^2^(SD = 3.64). The mean BMI for the males was 27.84 kg/m^2^ and 24.42 kg/m^2^ for females. Consistent with this BMI, the body fat percentage for 22 (59.52%) subjects was ≥25%. The mean body fat percentage was 28.63% (SD = 7.01). The mean body fat percentage for males and females was 29.46% and 26.82%, respectively. Waist circumference was ≥87 cm in 18 out of 21 males (48.65%) and ≥82 cm in 14 females (37.84%) out of the 16 screened. A waist circumference of >87 cm in males and >82 cm in females is suggestive of risk for type 2 diabetes.

The sagittal abdominal diameter was ≥21.5 cm in 21 subjects (56.72%) with a mean value of 22.2 cm (SD = 3.14). The SAD was found to be ≥21.5 cm in 14 males (66.66%) and in 7 females (43.75%). The SAD ≥ 21.5 cm indicates the risk for type 2 diabetes. The WHR was ≥0.89 in 15 males (40.54%) and ≥0.81 in 9 females (24.32%) with a mean value of 0.92 (SD = 0.07), suggesting a type 2 diabetes risk in majority of the subjects screened (see Table 2).

#### 4.1.3. Physical Activity and Fruits/Vegetable Intake

The physical activity pattern of 25 subjects (67.62%) showed an activity level of <3000 MET/week, indicating sedentary behavior. Most of the subjects (*n* = 24, 64.92%) reported consuming fruits and vegetables more than 3-4 times/week (see Table 2).

### 4.2. Relationship/Association among Measures

The relationship/degree of association of physical activity, length of immigration in US, family history of type 2 diabetes, and fruits and vegetable intake with the anthropometric measures and body fat percentage were analyzed. The analysis showed a relationship with physical activity of <3000 MET/week and (a) BMI ≥ 23 kg/m^2^ (*r* = 0.926, *P* = 0.032), (b) WC of >82 cm in females (*r* = 0.533, *P* = 0.066) and WC of >87 cm in males (*r* = 0.512, *P* = 0.062), (c) WHR of ≥0.81 in females (*r* = 0.467, *P* = 0.714) and WHR of ≥0.85 in males (*r* = 0.539, *P* = 0.082), (d) SAD of ≥21.5 cm (*r* = 0.32, *P* = 0.071), and (e) body fat percentage of ≥25% (*r* = 0.631, *P* = 0.041). The correlations were statistically significant for physical activity with BMI and body fat percentage.

Immigration time greater than 10 years in the US was associated with increased body fat percentage compared to other categories of immigration time in the US (*χ* ^2^ (4, *N* = 15) = 9.52, *P* = 0.041; Cramer's *V* = 0.34). It was further associated with BMI ≥25 kg/m^2^ (*χ* ^2^ (4, *N* = 3) = 10.24, *P* = 0.041; Cramer's *V* = 0.38). The analysis revealed that the degree of association was statistically significant for the immigration time greater than 10 years with body fat percentage and BMI ≥ 25 kg/m^2^. The immigration time over 10 years and other categories of immigration time in the US were not statistically significant with other anthropometric measures. Family history of type 2 diabetes was associated with increased body fat percentage, (*χ* ^2^ (2, *N* = 21) = 6.23, *P* = 0.041; Cramer's *V* = 0.34) and was statistically significant. Those who were reporting an increased consumption of fruits and vegetables consumption did not show a statistical significant association with the anthropometric measures and body fat percentage.

## 5. Discussion

The overall aim of this pilot study was to describe type 2 diabetes risk among Asian Indian adults of Kerala ethnicity living in the US. The analysis of BMI revealed that most of the women in this study were overweight and the men were obese. Asian Indians generally exhibit lower Body Mass Index and waist circumference and tend to accumulate intra-abdominal visceral fat when compared to Caucasians [12]. Studies report that BMI and waist circumference serve as parameters to estimate general or abdominal fat masses, respectively. The strength of association between waist circumference and type 2 diabetes risk depends on body mass index. Individuals of low or normal weight with a large waist circumference have the same risk of developing type 2 diabetes as preobese individuals with small waist circumference [29, 33]. The BMI and waist circumference were higher in most of the males and females screened in this study.

The sagittal abdominal diameter is a strong anthropometric marker of insulin resistance and hyperinsulinemia in obese men than the commonly used waist-to-hip ratio and other anthropometric measures. Sagittal abdominal diameter is a more independent measure compared with waist circumference to predict arterial stiffness in subjects with type 2 diabetes [34, 35]. Several common anthropometric measures correlate with body fat and abdominal fat. Obese individuals with excess visceral fat have an increased risk for the development of type 2 diabetes [8, 15]. The anthropometric measures and body fat percentage were higher in most of the subjects screened in this study, suggesting type 2 diabetes and cardiovascular disease risk in the Asian American Indians.

In a study that included Asian Indian immigrants living in the US, respondents had high physical inactivity but poor knowledge of cardiovascular disease risk factors [16]. The physical activity pattern of the majority of the subjects in our study showed an activity level of <3000 MET/week, indicating behaviors consistent with sedentary behavior. This study revealed that decreased physical activity was associated with higher BMI and body fat percentage in Asian Indians. This study finding was consistent with other studies focusing on physical activity patterns among Asian Indians living in the US [14, 15].

Studies have shown that adopting US norms and culture may lead to obesity and type 2 diabetes among immigrants as well as poor control of type 2 diabetes [36, 37]. The association between length of residence and the higher risk for obesity may be in part due to the adoption of poor dietary patterns and a sedentary lifestyle that is more typical of the host country [7, 8]. This study found that a length of residence in the US over 10 years was associated with an increase in the BMI and body fat percentage.

Family history has been shown to be a risk factor for the majority of chronic diseases such as type 2 diabetes and cardiovascular disease. Family history of diabetes is not only a risk factor, but it is also positively associated with risk awareness and risk-reducing behaviors [17, 18]. This pilot study revealed that a family history of type 2 diabetes was associated with higher body fat percentage.

The Diabetes Risk Score developed by Lindström and Tuomilehto [19] reported that consumption of fruits and vegetables less than 3-4 times/week predicts diabetes risk. The fruit and vegetable intake was more than 3-4 times/week for most of the subjects in the present study, indicating no predictable type 2 diabetes risk related to dietary pattern.

## 6. Limitations

This pilot study had the following limitations. It was conducted with a small sample of 37 Asian Indians of Kerala ethnicity living in a West Texas County. This county did not have many Asian Indian subgroups who met the inclusion criteria. A standardized type 2 diabetes risk assessment screening tool specific to Asian Indians living in USA has not been developed. Therefore, the reference values for the anthropometric measurements and body fat percentage are selected based on the recommendations from CURE Study [29], Indian Urban Based Study [30], and World Health Organization [28] criteria on obesity measures.

The 35-item Food Frequency Questionnaire (FFQ) developed by John Hopkins Weight Management Center measures various categories of individual dietary pattern. The reliability and validity of the FFQ has not been reported in the literature. Studies report that dietary recommendations for type 2 diabetes are focused mainly on relative dietary fat and carbohydrate content. Consumption of energy from protein at the expense of energy from either carbohydrate or fat contributes to disturbance of glucose metabolism and increased type 2 diabetes risks [9, 38]. The present study focused only on the consumption of fruits and vegetables intake per week to estimate type 2 diabetes risk [18]. 

The findings reflect the people of Kerala ethnicity, belonging to only one Asian Indian subpopulation, living in Texas. These immigrants settling in Texas and the South-West in general may have adopted eating habits consistent with the region; for example, eating calorie dense, deep fried foods, and red meat because of local influence. The dietary patterns among South Asians of Indian origin are variable and are also influenced by cultural/religious orientations. It is acknowledged that the sample does not represent all of South Asians/Asian Indians.

## 7. Conclusion and Recommendations

This pilot study used nonbiochemical measures of obesity, questionnaires to measure physical activity and fruits and vegetable intake, and self-report data on family history of type 2 diabetes, immigration time, history of hypertension, and fruit and vegetable intake to identify adults at risk for type 2 diabetes in Asian Indians of Kerala ethnicity living in a Southwestern Texas County of USA. The majority of subjects had higher indices of anthropometric measures and body fat percentage, placing them at risk for type 2 diabetes. The physical activity pattern of the majority of subjects showed a sedentary behavior. The physical activity level of <3000 MET/week was related to higher BMI and body fat percentage. Length of residence in the US greater than 10 years showed a significant association with body fat percentage and BMI. Family history of type 2 diabetes was associated with an increase in body fat percentage. The fruit and vegetable intake was adequate among the subjects and did not show an association with measures of type 2 diabetes risk.

Many associations between measures were not statistically significant. This could be due to the small sample size used in this pilot study failing to show true statistical significance or true statistical insignificance for those measures that did not show significance. However, the screening for type 2 diabetes risk and the emergence of the risk variables might have alerted subjects to their susceptibility for developing type 2 diabetes [27].

A number of recommendations result from this study. First, Asian Americans are an incredibly diverse population. Their family roots trace to the Far East, Southeast Asia, or the Indian subcontinent. A future study should include a larger sample representing Asian Indian subgroups from different regions of the US. Another need is the development of a population-specific diabetes risk score or validation of an existing tool for screening among a larger Asian Indian population living in US. For example, the Finnish Diabetes Risk Score (FINDRISC) is a feasible, noninvasive tool for identifying subjects at risk for undetected diabetes and prediabetes among Finnish population [39]. A tool such as this might be adapted for an Asian Indian population.

Third, national surveys report that Asian American/Pacific Islanders (AAPIs) are one of the fastest growing immigrant populations in the US in recent years. Prevalence of metabolic syndrome and associated risk factors are also reported to be high in Asian Indians living in the USA [8, 22, 24, 31]. Therefore, the present study recommends that more aggressive efforts should be directed toward screening Asian Indian subgroups for type 2 diabetes risks and implementing primary prevention approaches. 

## Figures and Tables

**Table 1:** Demographic variables.

Variables	*N*	%	Mean and SD
Age (years)			38.24 ± 16.24
20–29	13	35.12	
30–39	13	35.12	
40–49	2	5.44	
≥50	9	24.45	
Gender			
Male	21	56.76	
Female	16	43.24	
Religious preference			
Christian	37	100	
Others	—		
Food preferences			
Nonvegetarian	33	89.18	
Vegetarian	2	5.40	
Others	2	5.40	
Immigration time (in years)			
1–5	17	45.94	
5–10	5	13.54	
	15	40.52	
Family history of type 2 diabetes			
No	16	43.26	
Yes	21	56.82	
History of hypertension (mm of Hg)			
Normal: <120/80–90	28	75.75	
Prehypertension and hypertension: ≥120–140/90	9	24.32	
Physical activity (MET/week)			2338.38 ± 1505.53
Normal: >3000	12	32.42	
Sedentary: <3000	25	67.62	
Fruit and vegetable consumption			
Normal: >3-4 times/week	24	64.92	
Type 2 diabetes risk: <3-4 times/week	13	35.14	

**Table 2:** Type 2 diabetes risk categories for anthropometric measures and body fat percentage.

Variables	*N*	%	Mean and SD
BMI (kg/m^2^)			
Normal: <23	5	13.52	26.19 ± 3.64
Overweight: 23–25	14	37.83	
Obese: >25	18	48.64	
BMI			
Male	21	56.76	27.84 ± 3.26
Female	16	43.24	24.42 ± 5.26
Body fat percentage			
Normal: <25	15	40.56	28.63 ± 7.01
Type 2 diabetes risk: ≥25	22	59.52	
Male (≥25)	21	56.76	29.46 ± 2.36
Female (≥25)	16	43.24	26.82 ± 4.12
Waist circumference (cm)			
Male			
Normal: <87	3	8.10	92.05 ± 9.94
Type 2 diabetes risk: ≥87	18	48.65	
Female			
Normal: <82	2	5.41	84.25 ± 718
Type 2 diabetes risk: ≥82	14	37.83	
Sagittal abdominal diameter (cm)			
Normal: <21.5	16	43.24	22.22 ± 3.14
Type 2 diabetes risk: ≥21.5	21	56.72	
Male (≥21.5)	14	66.66	22.61 ± 3.22
Female (≥21.5)	7	43.75	21.84 ± 3.34
Waist-hip ratio			
Male			
Normal: <0.89	6	16.22	0.92 ± 0.07
Type 2 diabetes risk: ≥0.89	15	40.54	
Female			
Normal: <0.81	7	18.92	0.86 ± 0.17
Type 2 diabetes risk: ≥0.81	9	24.32	
